# Understanding the acute and chronic effects of basketball dual-task complexity on athletes’ inhibitory capacity

**DOI:** 10.1038/s41598-026-44305-x

**Published:** 2026-04-13

**Authors:** Alejandro Gutiérrez-Capote, Jesús Jiménez-Martínez, Francisco Alarcón, Anthony S. Leicht, Elisa Torre, Jose J. López-Morales, Iker Madinabeitia, David Cárdenas

**Affiliations:** 1 Department of Physical Education and Sport, Faculty of Sports Science, University of Granada, Granada, 18018 Spain; 2 Sport and Health University Research Institute (iMUDS), Granada, 18007 Spain; 3 Department of General and Specific Didactics, Faculty of Education, University of Alicante, Alicante, 03690 Spain; 4 Sport and Exercise Science, James Cook University, Townsville, QLD 4811 Australia; 5 Australian Institute of Tropical Health and Medicine, James Cook University, Townsville, QLD 4811 Australia

**Keywords:** Executive function, Cognition, Cognitive control, Dual-tasking, Adaptation, Sports, Health care, Neuroscience, Physiology, Psychology, Psychology

## Abstract

Inhibitory control (IC) enables athletes to suppress inappropriate responses and shift attention during sport practice. However, how acute training effects on IC relate to chronic adaptations remains unclear. This study investigated whether cognitive-motor complexity during interventions fosters long-term adaptations and how IC capacity moderates outcomes. One hundred twenty-five healthy athletes (68 men, 57 women; mean age, 21.1 ± 1.8 years) were divided into high- and low-IC capacity groups and assigned to one of four basketball cognitive motor dual-task interventions, varying in executive demands and physical load. A repeated-measures design assessed the effects of 11 training sessions on IC and whether the effects remained after three months. High-capacity groups showed improved IC accuracy and response time in each session (*p* < 0.001), followed by low-capacity groups, initially under low executive demands (*p* < 0.001) and then high executive demands (*p* < 0.001). At three months, performance generally declined (*p* < 0.001), except in the high-capacity group under high-demand conditions, where it was maintained. Notably, the negative performance peak during interventions correlated with final IC assessment (*p* = 0.002). Cognitively demanding training enhanced IC in athletes with a higher baseline capacity, while less complex tasks were more effective for individuals with lower capacity. These findings support adapting task complexity to cognitive level to optimise cognitive development.

## Introduction

Intervention studies examining the effects of physical exercise or sports on executive functions have generally reported both short-term and long-term benefits^1^. Executive functions are high-order cognitive capacities that support goal-directed behaviour, with inhibitory control (IC) playing a central role by suppressing inappropriate responses and enabling flexible adaptation in dynamic environments^2^. The ability to halt planned movements is essential for preventing errors and adapting actions in complex environments based on incoming information^3^. Greater IC capacity is linked to self-regulatory skills essential for learning and neural efficiency related to sports performance^4^.

However, not all types of physical exercise provide the same benefits for the development of executive functions. Specifically, acute effect studies incorporating sports practices have shown mixed findings for IC, with some reporting improvements (e.g. Ludyga et al.^5^) and others finding no significant effects (e.g. Lind et al.^6^). Recent work has even documented both positive and negative IC effects, depending on the mental load induced by constraints in one-on-one basketball tasks^7^. Subsequent evidence indicates that these acute responses are moderated by baseline IC capacity, mitigating the effects of cognitive load and performance decline during the tasks^8^. Evidence from chronic interventions is also heterogeneous, ranging from positive outcomes (e.g. Cho et al.^9^) to minimal or null effects (e.g. Condello et al.^10^). A recent meta-analysis confirmed these discrepancies, highlighting substantial heterogeneity in the observed effect sizes across both acute and chronic studies (see Lubans et al.^11^). These inconsistencies likely reflect the lack of standardised criteria, methodological diversity across studies^12^, and the limited consideration of motor complexity in task design^13^, all of which obscure mechanisms and applications.

Given the lack of consensus, research has shifted towards identifying the dose of exercise, emphasising both quantitative and qualitative aspects^1^, and examining the potential moderators such as individual characteristics^14^ or the level of physical exertion associated with task execution^15^. The Cognitive Stimulation Hypothesis is one of the most widely used frameworks to explain cognitive improvements after a single physical exercise session^16^. This hypothesis suggests that the type and intensity of the activity, especially its complexity and cognitive challenge, moderate the effects, such that the greater cognitive demand may heighten stimulation and yield cognitive and neural benefits. Yet, findings testing this proposition remain mixed and at times divergent^17^.

To address this conceptual ambiguity, the present work clarifies and operationally defines the main constructs under study. In the present work, physical exertion has been defined as the physiological effort associated with task performance, operationalised through heart rate^15^. Task complexity refers to the coordination and decision-making challenges imposed by basketball tasks, operationalised via constraint manipulations such as the number of response options and environmental uncertainty^18,19^. Cognitive demand is defined as the mental effort required to manage interference and adapt responses under time pressure, directly linked to the load on IC^2^. In line with the Challenge Point Framework proposed by Guadagnoli and Lee^20^, it is essential to distinguish between task complexity and task demand. Task complexity represents a nominal characteristic of the task, defined by the number of informational elements, response alternatives, and coordination requirements that must be monitored for effective task performance, and therefore remains independent of the individual performing the task. In contrast, task demand reflects functional task difficulty, emerging from the interaction between these nominal task characteristics (including task complexity) and the performer’s skill level, experience, and current cognitive state. Accordingly, increases in task complexity are expected to elevate task demand. However, the magnitude of this demand varies across individuals depending on their baseline cognitive capacity.

In the present study, task complexity was systematically manipulated as a nominal task property through changes in cognitive–motor constraints, whereas cognitive demand corresponded to the functional challenge imposed on athletes, indexed through their acute IC responses.

Recent evidence suggests that the association between cognitive demand and executive control may follow a curvilinear pattern^21^, where insufficient demand under-engages executive systems, intermediate, optimally challenging demand elicits benefits, and excessive load can overwhelm resources, impairing performance^22^. Given the established links between executive functions and sports performance^23^, systematically manipulating task complexity provides a principled approach to examining how athletes respond to the cognitive-motor demands imposed by the task and whether baseline cognitive capacity moderates these responses.

Despite these advances, the current evidence is insufficient to comprehend the relationship between the acute effect of a physical exercise session and the cumulative chronic effect of repeated practice, including the direction of these effects, either positive or negative. To advance this field, it is essential to monitor acute training responses across extended interventions, not only in the physical and motor domains but also in the cognitive domain^24^, as this may yield valuable and innovative insights for both researchers and practitioners.

To conceptually frame these ideas, the present study integrates the Cognitive Stimulation Hypothesis^16^ with the Challenge Point Framework proposed by Guadagnoli and Lee^20^. The Challenge Point Framework was initially developed in the field of motor learning and was not designed to predict cognitive load or cognitive enhancement directly. In this context, it is used conceptually to guide the interaction between motor task complexity and performer skill level, producing a functional challenge that engages executive processes^25,26^. Converging evidence shows that activities requiring complex motor coordination and high cognitive load more strongly engage neural circuits, particularly prefrontal regions, implicated in executive functions, than tasks involving minimal interference or uncertainty^27^.

Further support for this integrated approach comes from cognitive-motor dual-task (CMDT) paradigms, which require participants to manage simultaneous motor and cognitive demands^28,29^. Although they often induce immediate performance decrements, they can lead to long-term gains in both motor and cognitive domains^30^. Moreover, long-term CMDT interventions have been shown to promote cognitive and neural adaptations associated with improved decision-making^31^, supporting the relevance of this training approach for enhancing executive functions. Exposure to diverse contexts helps reveal how individuals allocate cognitive resources to adapt their performance for each task^32^, enabling analyses of how cognitive load affects skill execution and how interventions influence the development of executive functions over time^33^. Consequently, it is critical to monitor acute training responses across extended interventions, not only in physical and motor domains but also in the cognitive domain, to illuminate the temporal dynamics of adaptation^24^.

### Study aims and hypotheses

Building on this framework and addressing the identified research gap, the present study investigates how acute and chronic effects of physical exertion interact when cognitive-motor task complexity is systematically manipulated in basketball. Specifically, this research aims to: (O1) determine whether the acute effect of basketball tasks on IC varies with their cognitive-motor task complexity; (O2) analyse the relationship between the direction of the acute effect (positive or negative) and the cumulative (chronic) effect; and (O3) assess whether differences in baseline cognitive capacity moderate these outcomes. We hypothesise that: (1) increasing task complexity will differentially modulate post-exercise inhibitory capacity; (2) initial adverse acute effects on inhibitory capacity will precede positive adaptations observed across the six-week intervention period, with induced effects being maintained at the three-month follow-up; and (3) baseline cognitive capacity will moderate training-related changes in inhibitory capacity.

## Materials and methods

### Ethics committee approval

This study received ethical approval from the University of Granada Ethics Committee (No. 3618/CEIH/2023) and was conducted in accordance with the Declaration of Helsinki. Participants received detailed information about the study and provided written informed consent prior to participation.

### Experimental design

The study employed a repeated-measures mixed design, conducted over six months, comprising four types of CMDT interventions. The aim was to examine how different levels of task complexity, induced through basketball dribbling-based CMDTs, affected IC over time. Baseline inhibitory capacity was statistically controlled as a potential moderator of the intervention effects.

**Fig. 1:**
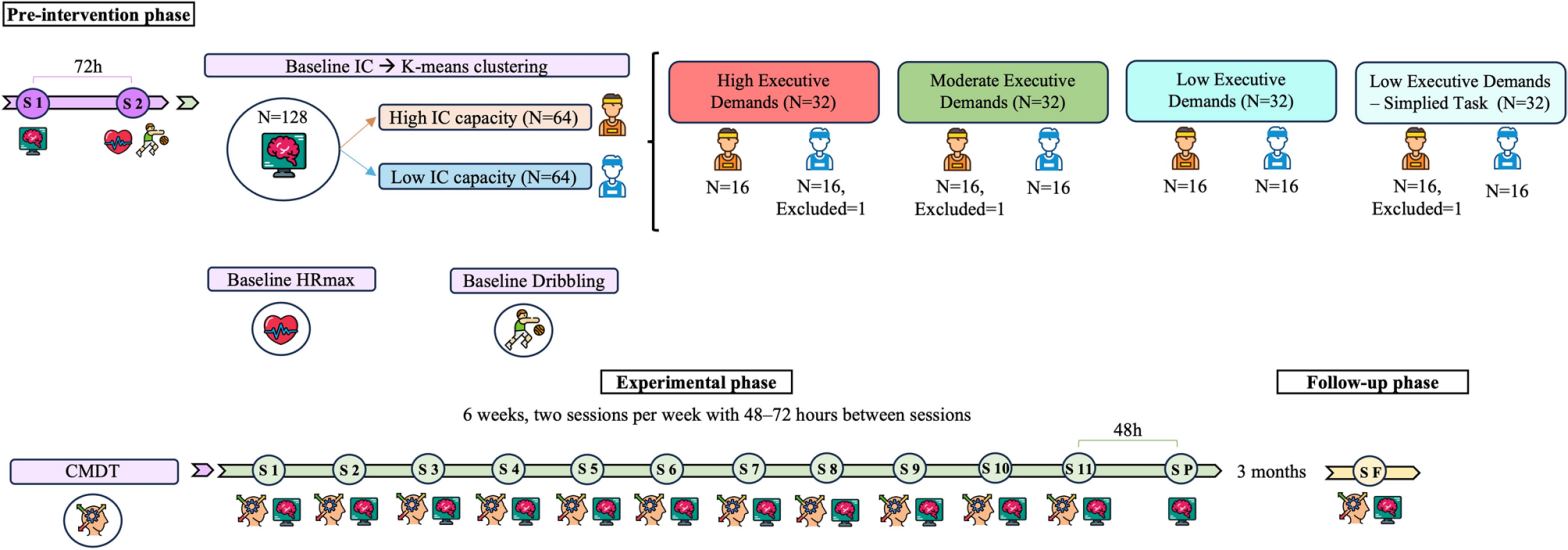
Display of the full experimental design, outlining the main phases and emphasizing the role of the pre-intervention phase in baseline control of participant characteristics and group allocation. S: Session; N: Number of included participants; IC: Inhibitory control; CMDT: Cognitive–motor dual-task intervention; HRmax: Maximal heart rate; h: hour(s).

The experimental protocol consisted of three consecutive phases (Fig. 1): (a) pre-intervention phase including participant recruitment and baseline assessments of IC and motor performance; (b) an experimental phase lasting six weeks involving four CMDT intervention types, with repeated IC assessments conducted after each training session to capture (1) acute effects, (2) possible detection of supercompensation between sessions (i.e., transient changes in IC performance between sessions across the intervention), and (3) the chronic effect observed after the final session. In addition, a post-intervention reassessment was performed 48 h after completing the intervention; and (c) a follow-up phase was conducted three months later to determine whether the training-induced effects were maintained over time.

Three of the four CMDT interventions implemented in the study combined a sport-specific motor skill, such as basketball dribbling, with simultaneous real-time processing of external visual stimuli^34^. In these conditions, cognitive–motor complexity was manipulated through different levels of executive demand (high, moderate, and low), generated by altering the amount of sensory information and the number of stimulus–response rules during task execution^8^. In the low executive demand condition, executive demand was minimised using a simple and predictable oddball paradigm, allowing the condition to serve as a control reference for comparison with interventions involving higher levels of cognitive–motor complexity^8,35^. Across these conditions, physical load was standardised between 80% and 90% of maximum heart rate (HRmax) in order to replicate the physiological intensity typically observed in basketball^36^ and to ensure a comparable physical load across CMDT conditions.

A fourth CMDT condition was included, corresponding to a seated visuomotor task performed at a table. This condition excluded both dribbling and physical exertion and involved minimal executive demands implemented through an oddball paradigm. It served as a control condition with very low cognitive–motor engagement, allowing isolation of attentional processing under minimal cognitive–motor demand.

Based on this design, four CMDT conditions were defined (Table 1): (1) High Executive Demand (HED); (2) Moderate Executive Demand (MED); (3) Low Executive Demand (LED); and (4) Low Executive Demand – Simplified Task (LED–S).

**Table 1:** Characteristics and manipulations of the Cognitive–Motor Dual-Task (CMDT) intervention conditions.

Condition	Task complexity	Executive demand	Physical load (%HRmax)
High executive demand	Dribbling task	Multiple visual stimuli and frequent updating of stimulus –response rules	80–90%
Moderate executive demand	Dribbling task	Moderate stimuli and fixed rules	80–90%
Low executive demand	Dribbling task	Oddball sequenced (predictable visual stimuli)	80–90%
Low executive demand – Simplified task	Seated visuomotor task	Oddball sequenced (predictable visual stimuli)	—

Note: The table summarizes the key manipulations applied across CMDT conditions. HRmax: Maximum heart rate.

### Participants

An a priori power analysis was conducted in G*Power (3.1.9.7)^37^ for a repeated-measures ANOVA with between-subject factors to determine the required sample size and ensure adequate representation of participants with both high and low capacity of IC across all CMDT experimental conditions. The analysis assumed a moderate effect size (f = 0.35), statistical power (1–β) = 0.95, an intra-class correlation coefficient (ICC) = 0.50, and α = 0.05, resulting in a minimum required sample size of 104 participants.

To ensure sample comparability and participant safety, inclusion criteria required participants to be federated university athletes actively engaged in open-skill sports (e.g., basketball, soccer, handball), completing at least 270 min per week of structured training and regular competition. This athletic profile was selected to ensure sample homogeneity and to control for variability in motor coordination and task familiarity that could otherwise confound cognitive outcomes. Previous research has shown that athletes from open-skill disciplines exhibit superior IC, attentional flexibility, and decision-making efficiency compared with non-athletes^4,38^. Additional inclusion criteria required: (a) absence of cardiovascular, neurological, or psychiatric disorders; (b) no use of medication affecting the central nervous system; (c) no recent concussion; and (d) no musculoskeletal injury or surgery within the past year.

A total of 128 healthy university athletes were recruited (69 males, 59 females). Participants (*n* = 128) were classified based on their baseline IC performance (Fig. 1). A K-means cluster analysis was conducted on baseline IC performance data (accuracy and response time) using SPSS v23. The number of clusters was set to k = 2: high-capacity (*n* = 64) and low-capacity (*n* = 64). Each capacity group was then randomly divided into four subgroups of 16 participants (i.e., high capacity: 16–16–16–16; low capacity: 16–16–16–16), resulting in a total of eight groups. These subgroups were subsequently assigned to one of the four intervention conditions. Due to dropouts and failure to complete all intervention sessions, the final sample was reduced to 125 participants. Table 2 summarises participant allocation, demographic characteristics, and baseline motor performance measures for the final sample.

**Table 2:** Participant allocation, demographic characteristics, and baseline motor performance measures for the final sample. Values are presented as mean (± SD).

Outcome variable	High executive demand		Moderate executive demand		Low executive demand		Low executive demand – Simplified task		All
	High IC group	Low IC group	High IC group	Low IC group	High IC group	Low IC group	High IC group	Low IC group	
N (M/F)	9/7	9/6	8/7	8/8	8/8	9/7	8/7	9/7	68/57
Age (yr)	21.9 (± 1.9)	21.1 (± 1.4)	21.3 (± 1.5)	21.2 (± 1.4)	20.9 (± 2.0)	20.0 (± 2.4)	20.9 (± 2.2)	21.2 (± 1.1)	21.1 (± 1.8)
Height (m)	1.77 (± 0.07)	1.75 (± 0.10)	1.75 (± 0.09)	1.74 (± 0.08)	1.73 (± 0.08)	1.74 (± 0.09)	1.72 (± 0.08)	1.76 (± 0.09)	1.75 (± 0.08)
Body mass (kg)	74.0 (± 11.6)	70.9 (± 9.1)	70.2 (± 8.2)	70.3 (± 10.9)	72.1 (± 11.5)	68.6 (± 11.9)	66.9 (± 8.7)	70.6 (± 8.8)	70.5 (± 10.1)
BMI (kg/m^2^)	23.7 (± 2.6)	23.1 (± 2.3)	22.9 (± 1.7)	23.0 (± 2.7)	23.8 (± 2.1)	22.6 (± 2.3)	22.6 (± 1.6)	22.8 (± 2.5)	23.1 (± 2.3)
Sport experience (yr)	9.3 (± 1.3)	9.6 (± 0.8)	9.5 (± 1.9)	9.6 (± 1.1)	9.9 (± 1.7)	9.4 (± 1.4)	9.2 (± 1.5)	9.3 (± 1.2)	9.5 (± 1.3)
Maximum heart rate (bpm)	197.6 (± 3.6)	196.8 (± 4.7)	200.9 (± 4.7)	197.4 (± 3.7)	197.0 (± 4.8)	196.1 (± 3.6)	199.1 (± 4.6)	199.3 (± 3.8)	198.0 (± 4.4)
Dribbling test 1 (s)	9.3 (± 1.2)	9.6 (± 2.3)	9.9 (± 1.8)	10.8 (± 1.9)	10.2 (± 1.4)	10.9 (± 1.9)	10.2 (± 1.2)	10.4 (± 2.0)	10.2 (± 1.8)
Dribbling test 2 (s)	12.0 (± 1.8)	12.3 (± 2.1)	12.4 (± 2.4)	12.9 (± 1.5)	12.5 (± 1.8)	13.1 (± 2.0)	12.6 (± 1.4)	13.2 (± 2.5)	12.6 (± 1.9)
Dribbling test 3 (s)	11.0 (± 1.8)	12.0 (± 3.7)	12.9 (± 4.7)	14.0 (± 4.5)	12.6 (± 2.8)	13.7 (± 2.5)	13.7 (± 3.9)	13.1 (± 3.0)	12.9 (± 3.5)
Dribbling test 4 (s)	14.3 (± 2.5)	15.3 (± 3.6)	15.1 (± 4.1)	17.1 (± 3.7)	15.2 (± 2.7)	15.6 (± 2.3)	15.4 (± 2.8)	15.4 (± 3.0)	15.4 (± 3.1)
Dribbling test 5 (s)	14.1 (± 3.1)	17.1 (± 4.0)	16.8 (± 5.4)	19.7 (± 5.4)	17.0 (± 5.4)	19.7 (± 5.3)	18.1 (± 5.0)	18.2 (± 5.7)	17.6 (± 5.1)

Note: IC: Inhibitory control; BMI: Body mass index; yr: years; N: Number of participants; M: Male; F: Female; kg: kilograms; m: meters; bpm: beats per minute; s: seconds. Dribbling tests 1–5: (1) simple front hand switch, (2) double front hand switch, (3) between-the-legs switch, (4) front hand and between-the-legs switch, and (5) between-the-legs and behind-the-back switch.

### Procedure

The study lasted six months and included 17 sessions. Two preliminary sessions were conducted before the experimental phases, separated by 72 h. The first was a one-hour information session explaining the study objectives, participation requirements, and informed consent procedures. The second was a two-hour familiarisation session during which participants submitted their signed consent forms and acclimatised to the laboratory setup. Participants were instructed to comply with standardised conditions^7^: avoid strenuous exercise for 48 h, alcohol for 24 h, and caffeine for 12 h, while ensuring food intake within 3 h and at least 7 h of sleep. Compliance was verbally confirmed at the start of each session.

#### Pre-intervention phase

This phase assessed and controlled individual differences that could moderate the effects of the intervention. It consisted of two sessions 72 h apart: (1) a 30-minute baseline IC assessment was for group classification; and (2) a 1.5 h, motor assessment, including anthropometry, the 20 m Shuttle Run Test^39^ to estimate HRmax and define individual training zones (80–90% HRmax), and a standardised basketball dribbling test to control for technical proficiency^31^.

#### Experimental and follow-up phases

The six-week experimental phase consisted of twelve sessions (eleven intervention sessions and one final IC assessment). Participants attended the laboratory twice weekly, with 48–72 h rest between sessions at consistent times to minimise circadian variability^40^. The final IC assessment took place 48 h after the last intervention to minimise the effects of acute fatigue.

The follow-up phase, conducted three months later, replicated the participant’s assigned CMDT condition to assess the persistence of training effects while participants maintained their usual sports routines.

#### Cognitive motor dual-task setup

A FITLIGHT Training System (Model FL2201SLC, Canada) was used to create the CMDT environment. Each device contained internal and external lights that emitted different colours, cueing specific motor responses based on preset rules. Response timing was captured through photocells, and all data were transmitted to a real-time monitoring application that allowed precise control of stimulus presentation and task execution. The application recorded trial-by-trial performance data, from which two primary variables were extracted: (a) response accuracy, calculated as the proportion of correct responses relative to the total number of trials (range: 0–1); and (b) mean response time (milliseconds), computed for each session.

**Fig. 2:**
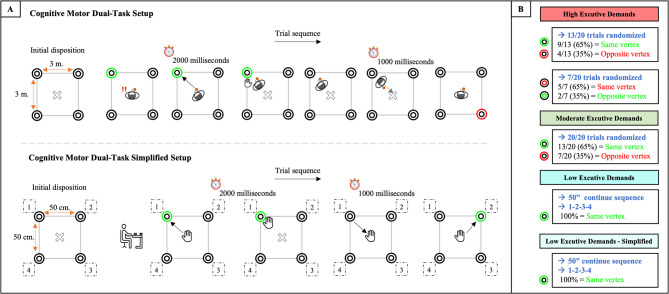
Graphical representation of the Cognitive–Motor Dual-Task (CMDT) setups used in the interventions. (A) Structure of the two task setups and an example of a trial sequence within a 50-second work interval, illustrating the temporal order of stimulus presentation and responses. (B) Manipulation of executive demand per intervention condition across intervals, based on the number of stimulus–response rules (responding to the same or opposite illuminated vertex) and the proportion of trials assigned to each rule within the different CMDT conditions.

In the conditions involving basketball dribbling (HED, MED, and LED), participants performed the task within a 3 × 3 m square, with one device positioned at each corner and a marked central position (“X”) indicated on the floor (Fig. 2A). Participants were instructed to dribble the ball continuously within the designated area while remaining at the marked central position (“X”) and, when one of the four devices changed from its neutral visual state (black light) to an activated state (illuminated), to move toward the corresponding corner to deactivate it, subsequently returning to the centre to await the next stimulus. These instructions were provided during the familiarisation session, during which participants completed three practice blocks of 10 trials using the same stimulus–response structure as in the experimental sessions to ensure correct task execution. Participants were also informed that responses would be considered incorrect if they failed to return to the centre between stimuli or initiated movement before the light was activated. Each session consisted of three blocks of five 50-second work intervals, separated by 10-second rests and one-minute breaks between blocks, ensuring an intensity of 80–90% of HRmax^41^.

In the LED–S condition, participants performed the task seated in front of a table on which a 50-cm square area was presented, delimited by a device at each vertex (Fig. 2A). The exact layout and timing as in the other conditions were maintained, but without dribbling or physical exertion.

### Executive demand manipulation

Executive demand complexity followed Gutiérrez-Capote et al.^8^, with adjustments to the frequency and spatial location of stimuli (Fig. 2B). In the HED and MED conditions, colour–position associations varied across devices, requiring participants to update stimulus–response mappings. In the HED condition, two stimulus–response rules were applied: (1) External lights: if a green light was activated, participants moved while dribbling the ball toward the same device to deactivate it; if a red light was activated, they moved toward the opposite device. (2) Internal lights: if a red light was activated, participants moved toward the same device; if a green light was activated, they moved toward the opposite device. Of the 20 trials presented per interval in each block, 65% followed Rule 1 (13 trials) and 35% followed Rule 2 (7 trials). Within each rule, 35% of trials required movement toward the opposite device (5 out of 13 trials in Rule 1 and 2 out of 7 trials in Rule 2). In the MED condition, a single rule was applied. Participants responded only to external lights, moving toward the same device if the light was green and toward the opposite device if it was red. The trials were distributed as 65% to the same device and 35% to the opposite device.

Each block comprised 100 randomised trials (20 per 50-s interval) with a 2000-ms stimulus presentation and 1000-ms interstimulus intervals. In these conditions, to increase task complexity, the associations between light colour and response location were reversed between Block 1 and Block 2. For Block 3, in accordance with Xie et al.^42^, the rule-colour associations remained fixed for 6–10 trials before changing, requiring participants to infer the new rules based on the researcher’s feedback derived from their performance during the task: “correct”, “incorrect”, or “late” (response exceeding the 2000 ms limit).

In the LED and LED–S conditions, a fixed response rule (deactivation of a green outer light) was applied following a predictable activation sequence (1–2–3–4–1) to minimise cognitive load. The exact stimulus timing as in the other conditions was maintained during the 50 s (2000-ms stimulus duration and 1000-ms interstimulus interval), with the same configuration applied across all three practice blocks. To prevent lateralisation bias, numbering proceeded from left to right for right-handed participants and from right to left for left-handed participants.

The task rules corresponding to each condition were explicitly explained to participants, and during the experimental sessions, task execution was visually monitored by trained investigators, with all sessions video-recorded to allow verification of compliance.

#### Intervention procedures

**Fig. 3:**
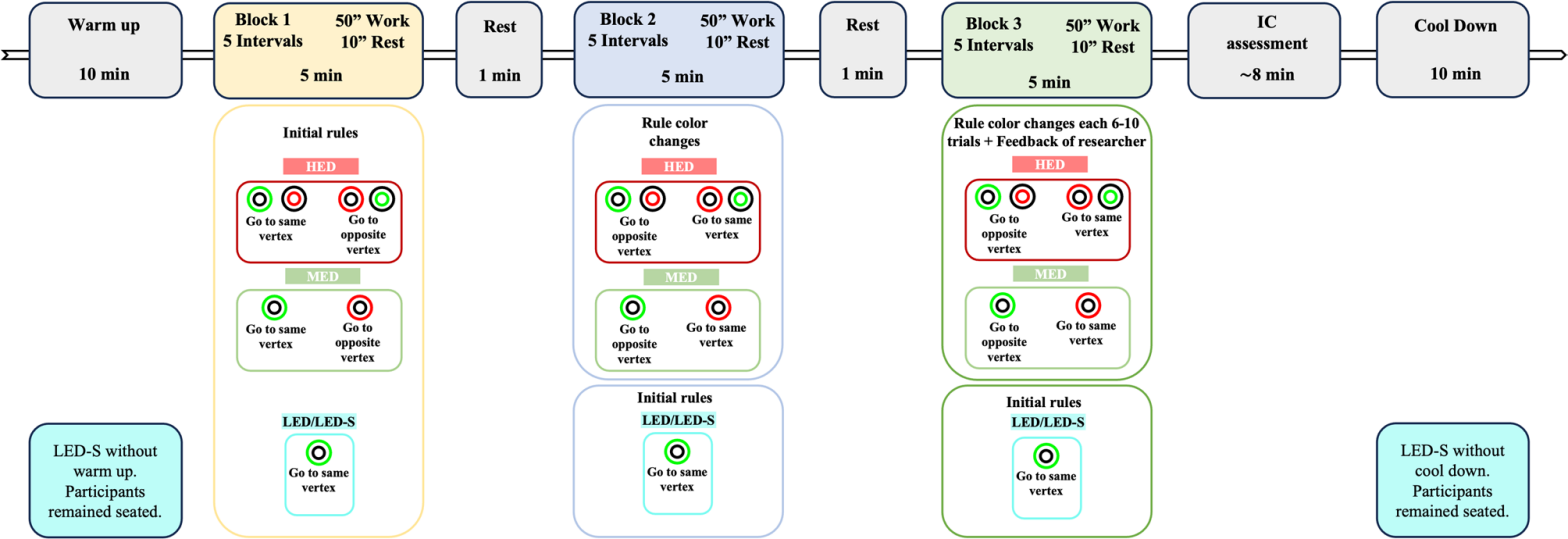
Overview of the experimental session timeline and CMDT progression, illustrating the sequence of work–rest intervals and the progressive manipulation of executive demand across blocks in the different intervention conditions. HED: High executive demand; MED: Moderate executive demand; LED: Low executive demand; LED–S, Low executive demand – Simplified task; IC: Inhibitory control; ”: seconds; min: minute(s).

Two intervention procedure types were implemented (Fig. 3). In the HED, MED, and LED conditions, participants wore a Polar H10 heart rate monitor (Polar Electro, Kempele, Finland). Sessions began with a 10-minute standardised warm-up (joint mobility, stretching, running, short sprints), followed by three practice blocks and an IC assessment (~ 8 min), and ended with a 10-minute cool-down.

In the LED–S condition, the protocol simulated that of the other interventions, except that the warm-up was replaced by a seated period to minimise cortical and muscular activation that could influence subsequent performance.

The same researcher supervised all sessions to ensure standardisation and adherence to the target intensity range.

#### Instruments

##### Inhibitory control assessment

IC was assessed using a Go/No-Go task^43^ implemented in SuperLab v.4.5 (Cedrus Corporation, San Pedro, CA, USA). The task was conducted in a soundproof, distraction-free classroom adjacent to the laboratory, equipped with a personal computer running Windows, a 27-inch monitor, a keyboard with a marked response key, and an adjustable chair. Participants sat 60 cm from the screen and responded to target stimuli following a fixed temporal sequence: a blank screen (500 ms), a central fixation cross (500 ms), a pre-stimulus cue (a black arrow; 1000 ms), and then the target stimulus. Participants had up to 1500 ms to respond by pressing the C key (“Go”) or by inhibiting their response (“No-Go”). Response rules were based on the orientation of the pre-stimulus arrow and the colour of the target stimulus, as follows: 1) For a downward arrow: (a) “Go” if it turned green and kept the direction; (b) “No-Go” if it turned turquoise green or reversed upwards; and 2) For an upward arrow: (a) “Go” if it turned green and kept the direction; (b) “No-Go” if it turned turquoise green or reversed downwards. The test comprised 180 randomised trials (122 “Go”, 67.78%; and 58 “No-Go”, 32.22%), lasting ~ 8 min. Performance indices included the mean response time (RT; in milliseconds) and the proportion of correct responses. Each session was supervised to ensure correct execution.

##### Anthropometric assessment

Height and weight were determined using a SECA 799 stadiometer and a digital scale (Seca, Germany; accuracy: 0.1 kg). Body Mass Index (BMI; kg·m²) was subsequently determined.

##### Dribbling skill

Dribbling skill was measured using a modified NBA Combine Test, validated as a reliable assessment method of basketball ability^29,31^. Conducted along the basketball court sideline, participants dribbled the ball at maximum speed, switching dribbling hand five times at 5.5-metre intervals marked by cones. Execution time was recorded with single-beam light cells (Chronojump BoscoSystem^®^, Barcelona, Spain) positioned 2 m apart and 1 m high (~ hip level). The test was completed ten times (five versions with two attempts each) with each test version using a different action as follows: (1) simple front hand switch; (2) double front hand switch; (3) between-the-legs switch; (4) front hand and between-the-legs switch; and (5) between-the-legs and behind-the-back switch. Starting 0.5 m behind the line in a two-leg stance, participants completed two attempts of each test version with a 2-minute rest, and the best time of each test version was recorded. Male participants used size seven basketballs, and females used size six basketballs for the test, as per international regulations.

##### Maximum heart rate determination and physical load control

Participants were equipped with a Polar H10 sensor and a Polar Pro chest strap (Polar Electro, Kempele, Finland) to monitor heart rate (HR) throughout the study. This system was synchronised with a Polar Vantage M smartwatch (Polar Electro, Kempele, Finland) operated by the researchers, allowing real-time monitoring of cardiovascular demands during the sessions. This ensured that each participant worked within the target intensity range (80–90% of HRmax) during the interventions. This method has proven effective for monitoring internal training load and its correlation with external load in basketball players^7,8^.

#### Statistical analysis

Descriptive statistics for the training load and IC variables are presented as mean ± standard deviation (SD). An ANOVA was conducted on the performance in the basketball dribbling test to determine if there were differences in motor skills between the groups. Subsequently, various mixed-effects analyses (Linear Mixed Models, LMM) were performed for the following objectives: (1) to verify that the training load (HRmax) during each training session was equal among the groups with physical load but different from those without load, and to verify the differences in cognitive task complexity among the four conditions; (2) to observe the effect of the intervention on IC, which involved constructing a model with the dependent variable being IC performance, the fixed factor was the group, and models were adjusted for prior performance (baseline or post-intervention) depending on the time point analysed, with participant as a random intercept; and (3) to analyse how IC performance varied across each intervention.

Finally, to examine the potential occurrence of an acute-to-chronic adaptation on IC, the percentage change for each participant was calculated as follows: ([post-training value − pre-training value] / pre-training value) × 100. Subsequently, a two-step analysis was conducted. First, a k-means clustering algorithm was applied based on the most pronounced negative performance peak observed across the 11 intervention sessions, categorising participants into two groups (i.e., higher vs lower). An independent samples analysis was then performed to assess differences in the final IC assessment between these groups. Second, a correlation analysis was conducted to explore the relationship between the magnitude of the negative performance peak and the final IC assessment. To simplify the analysis, only RT was used as the performance variable, as it was observed to exhibit more significant differences compared to the number of correct responses.

The significance level for all analyses was set at *p* < 0.05. Effect sizes were calculated using Hedges’ g, an unbiased measure of standardized mean differences. Interpretation followed conventional thresholds: small (0.2), medium (0.5), large (0.8), very large (1.2), huge (2.0), and gigantic (4.0 or greater). All analyses were performed using RStudio (version 2024.04.2). LMMs were fitted using the *lmer* function from the *lmerTest* package^44^. This package provides t-values and corresponding p-values for fixed effects using Satterthwaite’s approximation for degrees of freedom.

## Results

All data supporting these results are available in the open-access repository (https://osf.io/je34g/).

### Participants

No significant differences in participant characteristics (e.g., height, mass, HRmax, Basketball Dribbling Skill Test, and previous sports experience) were observed between groups.

### LMM - training results and complexity check

LMM was used to verify whether physical load was consistent across all groups. The model applied was: HRmax ~ Group + (1 | Participant). No significant differences for HRmax were observed between training sessions for intervention groups with physical load (84.9 ± 1.9% HRmax). Likewise, HRmax was similar across training sessions for groups without physical load (52.0 ± 1.4% HRmax), but significantly lower than in the intervention groups with physical load (t = − 50.95; *p* < 0.001; g = 10.96).

**Fig. 4:**
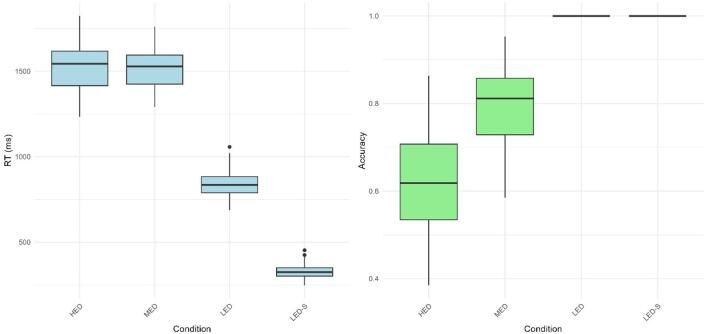
Differences in cognitive–motor dual-task intervention performance among the four conditions. HED: High executive demands; MED: Moderate executive demands; LED: Low executive demand; LED-S: Low executive demand - Simplified task; ms: milliseconds; RT: response time. * = *p* < 0.001.

Regarding the task complexity check, another LMM was performed following two different models. For accuracy, the model was: Accuracy ~ Condition + (1 | Participant); and for RT: RT ~ Condition + (1 | Participant). The mean performance in CMDT conditions across all sessions (Fig. 4) revealed that HED resulted in significantly lower accuracy compared to MED (t = -22.79; *p* < 0.001; g = -2.49). Furthermore, MED showed significantly lower accuracy than the LED conditions (t = -28.60; *p* < 0.001; g = -3.09). No significant differences were found between the two LED conditions. In terms of RT, no significant differences were observed between the HED and MED groups. However, both conditions exhibited significantly longer RT compared to the LED [vs HED (t = -41.93; *p* < 0.001; g = 9.79); vs MED (t = -41.94; *p* < 0.001; g = 9.79)], which, in turn, had a significantly longer RT than the LED-S (t = -31.51; *p* < 0.001; g = 7.35).

### LMM - intervention effects on IC

Significant differences were observed for the IC measures over time among the eight groups (Table 3). Regarding the number of correct responses, the model applied was Accuracy at post-intervention ~ Group + Accuracy at baseline + (1 | Participant). High and low capacity HED and MED groups demonstrated an increase at post-intervention compared to baseline, with the increase greater for the High capacity HED, High capacity MED and Low capacity MED groups compared to the Low capacity HED [Low capacity HED vs High capacity HED (t = -8.071; *p* < 0.001; g = 1.31); vs Low capacity HED (t = -4.84; *p* < 0.001; g = 1.16); vs High capacity MED (t = -2.81; *p* = 0.006; g = 2.31)]. At follow-up, the model applied was: Accuracy at follow-up ~ Group + Accuracy at post-intervention + (1 | Participant). Low capacity HED and MED and High capacity MED groups exhibited a reduction in correct responses compared to the post-intervention assessment (*p* = 0.028). No significant changes in the number of correct responses were observed for the High and low capacity LED and LED-S groups between baseline, post-intervention and follow-up assessments.

In terms of RT, the model used was: RT at post-intervention ~ Group + RT at baseline + (1 | Participant). High capacity HED and MED groups had the most significant improvement (-10.48% and − 8.23% respectively; t = 11.71; *p* < 0.001; g = -1.86), followed by the Low capacity HED (-3.91%; t = 8.10; *p* < 0.001; g = -0.37), Low capacity MED (-3.32%; t = 8.46; *p* < 0.001; g = -0.53), and Low capacity LED-S (-4.41%; t = 8.27; *p* < 0.001; g = -0.57). These groups outperformed the High capacity LED-S (-3.57%; t = 3.19; *p* = 0.002; g = -0.21), which in turn outperformed the High capacity LED (0.15%; t = 4.27; *p* < 0.001; g = 0.01), which outperformed the Low capacity LED (2.36%; t = 10.34; *p* < 0.001; g = 0.30). Performance three months later was analysed using the model: RT at follow-up ~ Group + RT at post-intervention + (1 | Participant). No significant differences for the High and low capacity HED and MED groups, while the other groups showed significant declines via longer RT (High capacity LED-S: 6.33%, t = 4.07, *p* < 0.001, g = 0.48; Low capacity LED-S: 5.58%, t = 8.52, *p* < 0.001, g = 0.46; High capacity LED: 8.72%, t = 4.18, *p* < 0.001, g = 0.46; Low capacity LED: 6.44%, t = 9.82, *p* < 0.001, g = 0.51).

**Table 3:** Inhibitory control (IC) outcomes comparing baseline–post-intervention and Session 11–follow-up assessments under Cognitive–Motor Dual-Task (CMDT).

Outcome variable	High executive demand		Moderate executive demand		Low executive demand		Low executive demand – simplified task	
	High IC group	Low IC group	High IC group	Low IC group	High IC group	Low IC group	High IC group	Low IC group
Correct responses								
Baseline measures (without CMDT exposure)								
BM	171.2 (± 3.5)	171.1 (± 2.1)	168.6 (± 2.3)	171.6 (± 2.6)	175.1 (± 2.1)	173.7 (± 2.9)	174.8 (± 3.3)	173.9 (± 2.9)
PM	175.4 (± 0.9)^**^	173.6 (± 2.4)^*^	173.1 (± 1.4)^*^	176.1 (± 2.1)^*^	175.3 (± 1.9)	173.6 (± 1.3)	174.3 (± 2.0)	173.3 (± 2.1)
Measures after CMDT exposure								
S11	173.8 (± 1.7)^**^	169.8 (± 1.2)	171.7 (± 1.7)	174.8 (± 2.9)	175.1 (± 3.4)	173.6 (± 3.2)	174.7 (± 2.7)	173.7 (± 2.0)
FM	174.6 (± 1.5)^†^	172.8 (± 2.4)^†^	168.9 (± 1.8)^†^	169.4 (± 1.6)^†^	174.9 (± 2.3)	173.4 (± 2.2)	174.0 (± 1.5)	173.9 (± 1.1)
Response time - correct responses (millisecond)								
Baseline measures (without CMDT exposure)								
BM	315.0 (± 15.6)	396.8 (± 47.3)	326.7 (± 21.7)	397.9 (± 28.8)	326.5 (± 41.3)	398.2 (± 34.8)	328.0 (± 61.5)	401.1 (± 35.4)
PM	282.0 (± 20.7)^**^	381.3 (± 17.8)^**^	299.8 (± 14.1)^**^	384.7 (± 23.4)^**^	327.0 (± 22.2)	407.6 (± 31.3)	316.3 (± 56.4)	383.4 (± 30.2)^*^
Measures after CMDT exposure								
S11	284.9 (± 25.4)	384.1 (± 22.0)	302.0 (± 18.1)	382.9 (± 30.9)	312.1 (± 55.9)	396.2 (± 46.7)	319.0 (± 46.0)	377.9 (± 47.7)
FM	295.5 (± 17.7)	390.6 (± 20.1)	308.6 (± 28.1)	392.9 (± 27.5)	339.3 (± 31.2)^†^	421.7 (± 26.6)^†^	339.2 (± 34.6)^‡^	399.0 (± 38.9)^†^

Note: BM: Baseline measure; PM: Post-intervention baseline measure; FM: Follow-up measure, assessed three months later; S11: IC measure obtained after completing the final CMDT training session (Session 11). *p < 0.05; **p < 0.001 (significant change: BM vs PM). †*p* < 0.05; ‡*p* < 0.001 (significant change: S11 vs MM).

### Acute-to-chronic adaptation in IC

**Fig. 5:**
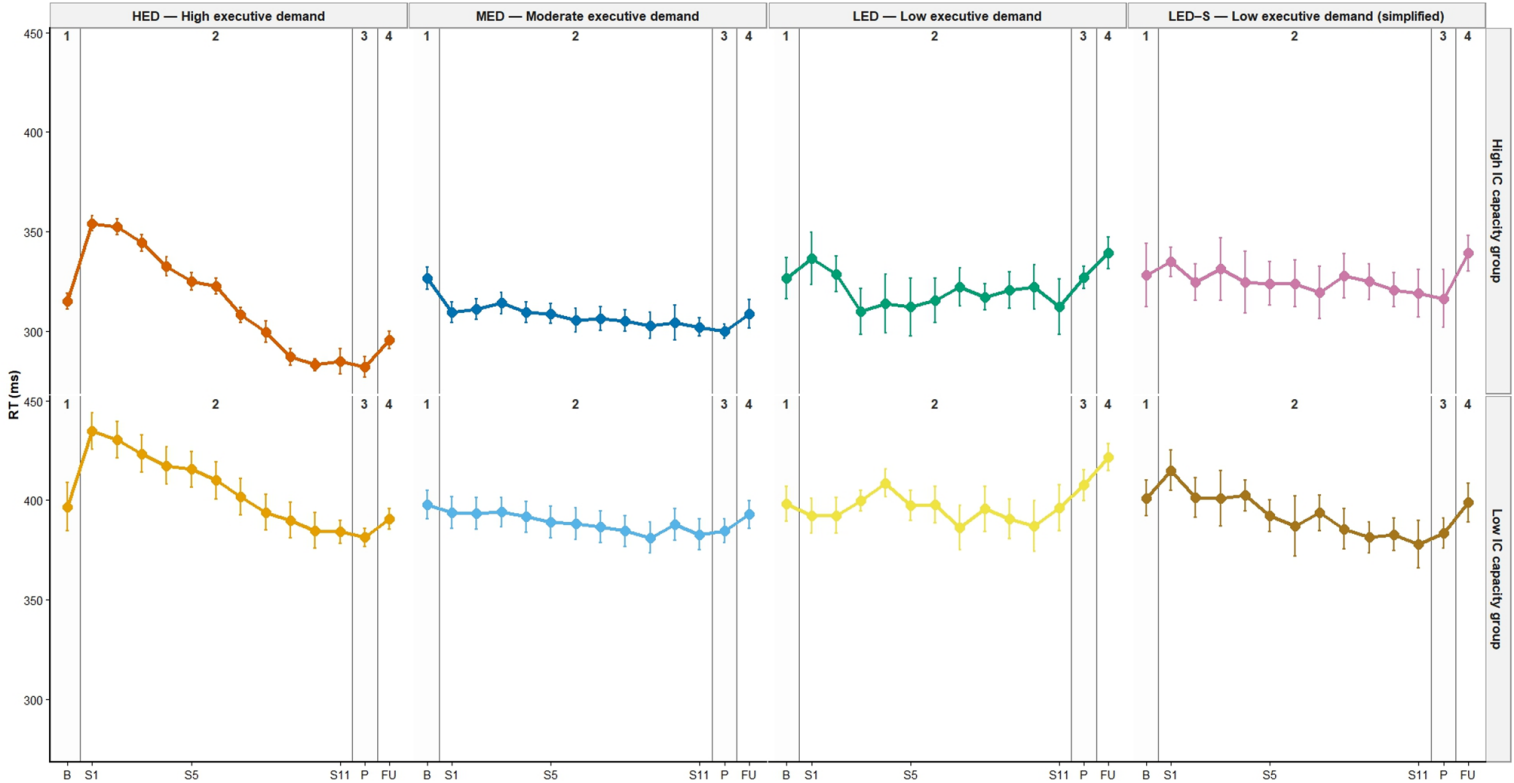
Group change in inhibitory control (IC) across the study. The first data point (1) corresponds to the baseline IC assessment, followed by performance data collected across 11 consecutive training sessions (2). The final two data points represent the post-intervention IC assessment (3) and the follow-up phase (4). Data points 1 and 3 reflect baseline performance measures, whereas data points 2 and 4 were recorded immediately after completing the cognitive-motor dual task. B: Baseline assessment; S1–S11: training sessions assessment 1 to 11; P: Post-intervention assessment; FU: Follow-up assessment; RT: Response time; ms: milliseconds.

The paired-samples analysis between the two cluster groups (t = 8.08; *p* < 0.001) indicated that individuals who exhibited a larger temporary worsening during training also demonstrated lower scores in the final IC assessment (i.e., slower RT). However, the correlation analysis conducted across the entire sample did not reveal any significant association between the most pronounced negative performance peak during the 11 training sessions and the final IC assessment. This means that the size of the worst temporary drop in performance during training did not reliably predict who would end up with better or worse IC in the overall sample. Notably, a significant inverse correlation was observed exclusively within the High capacity HED (rho = − 0.721; *p* = 0.002), consistent with an overload–adaptation pattern restricted to this subgroup. Figure 5 provides a visual representation of the change in IC performance.

## Discussion

The present study examined the acute and chronic effects of varying levels of CMDT complexity on IC in athletes. Overall, the findings revealed that experimental conditions with high cognitive-motor complexity (HED) led to lower accuracy in the short term than moderate-demand conditions (MED), while both MED and HED conditions resulted in chronic improvements in IC performance (Table 3). In contrast, the LED conditions failed to produce either acute or chronic effects, and a deterioration in RT was observed three months later. Moreover, athletes with higher baseline IC achieved greater long-term gains under high-demand conditions, whereas those with lower baseline capacity improved more under less demanding tasks. The absence of group differences in skill level or physical load supports the internal validity of the study, suggesting that the observed effects were primarily attributable to the manipulation of task complexity. Notably, only the high IC group in the HED condition exhibited an acute decline followed by chronic improvement, suggesting a pattern of cognitive adaptation in response to initial overload.

### Acute effects of task complexity

The acute findings confirmed that different levels of cognitive-motor demand had differential effects on IC performance. According to the cognitive stimulation hypothesis proposed by Pesce^16^, a task must reach a certain complexity threshold to generate cognitive benefits. Below this, stimulation is insufficient to activate adaptive processes, whereas excessive demand may exceed available resources, impairing performance through temporary overload^16^. In the LED conditions, cognitive load was minimal and limited to basic attentional processing without executive engagement. Even with high physical load, the tasks lacked sufficient cognitive-motor integration to induce adaptations. This aligns with previous findings indicating that low-stimulation tasks do not improve executive functions, especially when they lack a sufficient challenge in processes such as working memory or sustained attention^45^. This interpretation was supported by the inverted U-shaped relationship between stimulus intensity and cognitive performance, as observed in studies on the P3 wave^46^. This event-related potential component reflects the brain’s ability to allocate attentional resources and filter irrelevant information^46^. In LED conditions, minimal attentional demands were insufficient to significantly activate the P3. Since the required attention was clearly of low complexity, participants did not experience the need to filter irrelevant information, thereby limiting the recruitment of additional attentional resources. Hillman et al.^47^ emphasised that improvements in inhibition and cognitive flexibility were associated with tasks requiring sufficiently demanding cognitive processing, and that low-demand conditions failed to activate the prefrontal cortex or increase P3 wave amplitude adequately. Therefore, in the LED conditions, and considering the characteristics of the participants (active athletes in federated competitions), the cognitive demands may not have reached sufficient levels to generate significant effects, regardless of baseline IC.

For the MED conditions, tasks may have neared (but not met) the complexity threshold needed for adaptation. Although no significant gains emerged, these tasks required slightly more attentional and executive resources. This aligns with findings suggesting that minimal complexity can lead to specific improvements, such as faster processing speed, without broadly enhancing cognitive performance^48^. These tasks likely elicited mild prefrontal cortex activation, supporting moderate stimulation but insufficient for robust improvements^49^. Diamond and Ling^50^ noted that early gains may reflect initial engagement rather than lasting change, especially under limited cognitive stimulation.

In contrast, the HED conditions caused an IC acute decline. This effect was attributed to resource overload during complex task processing, leading to temporary depletion and impaired performance^22^. Similar results have been reported in multitasking studies, where excessive task complexity temporarily reduced accuracy and increased RT^8^. These tasks overcome the available resources (by exceeding the limits of the processing system), negatively affecting motor performance and decision-making accuracy^51^. Moreover, under high cognitive demand conditions, a deactivation in cortical areas linked to top-down behavioural control has been observed, hindering selective attention regulation and exacerbating the adverse effects on performance^31^. This decline was associated with increased perceived mental fatigue, which limited the ability to filter irrelevant information and maintain an adequate response accuracy^7,8^. The effort to sustain appropriate performance increases the requirement for attentional resources, leading to a progressive mental strain that contributes to the depletion of available resources^52^. These findings underscore the importance of surpassing minimum complexity thresholds to stimulate adaptive processes and guide the development of performance.

### Chronic adaptations and the role of cognitive thresholds

Long-term improvements in IC were determined by both task complexity and participants’ baseline inhibitory capacity. As expected, the LED conditions did not yield chronic benefits in IC immediately after the intervention. However, a notable increase in RT was detected during the follow-up session three months later, suggesting a delayed decline in performance. This lack of chronic benefits may be attributed to the limited cognitive stimulation provided by the oddball tasks, which likely did not surpass the minimum threshold needed to activate executive functions or trigger adaptive physiological responses^53^. However, the follow-up results indicated that although the minimal demands were insufficient to induce effects during the 11-session intervention, they may have helped prevent immediate deterioration by maintaining a minimal level of cognitive activation^48^.

In contrast, the MED and HED conditions revealed the positive impact of exposing athletes to more challenging stimuli, demonstrating both long-term benefits and the persistence of these effects over time (Table 3). These findings were consistent with previous studies, which have shown that physical exercise combining high cognitive demands with complex motor interactions supports the development of executive functions over time^10,54^. In this regard, activities involving greater cognitive-motor interaction appear more effective at activating neural circuits associated with cognitive control (especially in the prefrontal cortex and cerebellum), thereby promoting adaptive plasticity^27^. Additionally, intense physical activity combined with high cognitive effort has been shown to increase the release of brain-derived neurotrophic factor^55^. This factor plays a crucial role in brain plasticity by promoting the formation of new neural connections and enhancing the nervous system’s capacity to adapt to new challenges^55^. Recent research has documented cognitive improvements linked to increased brain-derived neurotrophic factor levels following both single exercise sessions^56^, and during prolonged sports training programs^9^. Notably, although the HED group initially exhibited cognitive fatigue and performance deterioration, it achieved the greatest long-term improvements (Table 3). This supported the view of Audiffren and André^57^, who argued that a high mental load can temporarily exceed self-regulatory capacity, but repeated exposure may lead to neuroadaptive responses that strengthen executive functions. This acute-to-chronic relationship was also observed by Moreira et al.^30^, whose findings showed that CMDT training initially impaired cognitive performance, yet sustained practice resulted in significant long-term gains. Similarly, Herold et al.^58^ suggested that cognitively demanding physical exercise may cause initial exhaustion followed by cognitive and emotional adaptations. With continued practice, initially complex tasks require fewer resources from the athlete, reflecting improved cognitive-motor efficiency^20,25,26^. The current results confirmed this relationship, as the acute depletions observed during high-complexity conditions were key in inducing significant chronic adaptations. Moreover, the results supported the notion that training complexity and associated cognitive demands that exceed an appropriate stimulation threshold can trigger positive adaptations in IC, with lasting and beneficial long-term effects.

### Interaction between task effects and baseline inhibitory capacity

Finally, our findings present an innovative perspective by highlighting the moderating role of initial IC capacity for intervention effectiveness. Previous studies have shown that high levels of IC reduce cognitive expenditure by suppressing inappropriate responses^4^, and that individuals with greater baseline cognitive resources perceive lower mental load and exhibit less deterioration in their motor and cognitive performance^8,59^. Our study extends this evidence by confirming that baseline IC not only influenced acute responses but also shaped sustainable adaptations under progressive demand conditions. The results indicated that all participants improved under both MED and HED conditions, regardless of initial cognitive ability (IC). While overall performance improved under both conditions, the analysis of RT revealed significant distinctions between participant profiles. Participants with low initial capacity who completed the high-demand conditions, despite experiencing an initial depletion of resources, showed significant improvements in RT by the end of the intervention, reaching levels comparable to those observed in low-capacity participants under low-demand conditions. These results were particularly notable among participants with high initial capacity exposed to high-demand conditions. Turakhia et al.^60^ noted that task adaptation based on individual capacities optimises the impact of experimental conditions. Our findings reinforce this perspective and confirm that, regardless of task complexity, considering individual capacities is essential for promoting sustainable improvements in cognitive and motor performance^14^.

This study offers a valuable perspective by highlighting that the effectiveness of sports interventions may largely depend on the alignment between task complexity and the individual characteristics of athletes. Furthermore, integrating both internal factors (such as baseline IC) and external ones (the specific demands of the task) may provide a more accurate framework for designing effective interventions. The current results support the notion that tasks positioned around the “optimal challenge point” foster sustainable adaptations, even if the initial effects were not always positive^1^. This would enable a better understanding of the acute mechanisms that trigger long-term adaptations^30^. The distinct linkage between the acute and chronic effects of exercise underscores the need for further studies to explore this area in greater detail. Identifying optimal challenge parameters would clarify the proper drivers of performance, enabling the design of interventions tailored to individual differences from the outset, and enhancing long-term adaptation and learning^60^.

### Limitations and future research

Although the present study was methodologically rigorous, several limitations should be acknowledged. While the sample size met the a priori power requirements, larger subgroup representation would enhance statistical precision and reliability. Additionally, since all participants were active university athletes engaged in open-skill sports, the generalizability of the findings to non-athletic populations or athletes from closed-skill disciplines remains limited. Moreover, although sport type and technical proficiency were controlled, residual transfer effects from other open-skill contexts cannot be excluded entirely. Although standardised pre-session guidelines were implemented to promote comparable testing conditions, adherence to these requirements ultimately relied on participants’ self-reported compliance, which cannot fully rule out minor deviations from the prescribed conditions. The study also relied exclusively on behavioural outcomes (accuracy and RT) without incorporating neurophysiological measures such as electroencephalography or event-related potentials, which could provide complementary evidence of cortical activation patterns. Finally, while the three-month follow-up period offered initial insights into the maintenance of effects, it does not clarify the long-term durability (beyond six months) of the observed adaptations. Future research should address these issues by incorporating neurophysiological correlates, larger and more heterogeneous samples, and longer longitudinal follow-ups to understand better how CMDT complexity modulates brain-behaviour adaptations^14^.

### Practical implications

The findings offer valuable guidance for practitioners designing cognitive–motor training programs in sports contexts. Coaches should align the level of dual-task complexity with each athlete’s IC capacity, progressively increasing cognitive load as the athlete adapts. In this process, introducing tasks that initially challenge and slightly exceed athletes’ IC capacity can stimulate short-term decrements followed by adaptive improvements, ultimately promoting more robust inhibitory functioning. Similarly, training strategies that incorporate unpredictable variability and changing stimuli may help athletes sustain cognitive engagement throughout practice, encouraging continuous adjustment of inhibitory responses to dynamic conditions. CMDT-based exercises can then be integrated into technical or tactical drills to improve athletes’ ability to manage interference and maintain focus under pressure. Finally, to ensure effective adaptation, coaches should monitor signs of cognitive fatigue during high-demand sessions, as excessive load may hinder both performance and learning efficiency.

## Conclusions

This study provides evidence that CMDT complexity influences both short- and long-term changes in athletes’ IC. High-demand CMDT conditions initially impaired performance but ultimately led to greater long-term improvements, whereas lower-demand tasks facilitated gradual yet smaller gains.

Notably, individual differences in baseline inhibitory capacity moderated these effects, as athletes with higher cognitive capacity responded more effectively to high-complexity training. In comparison, those with lower capacity benefited more from simpler tasks.

These findings underscore the importance of tailoring cognitive-motor task complexity to the athlete’s current cognitive profile to optimise adaptation (in line with the optimal challenge point framework^20^. Although the present results suggest that appropriately challenging dual-task training may enhance executive performance in sport, they should be interpreted cautiously given the study’s sample characteristics and behavioural focus.

Further research using neurophysiological measures and diverse athletic populations is warranted to confirm these mechanisms and refine evidence-based training guidelines.

## Data Availability

The database and R code used for the analyses are openly available at the Open Science Framework (OSF) repository: https://osf.io/je34g/.
